# Effect of biochar and mineral fertilizer integration on growth and nutrient dynamics of rice varieties under dry direct-seeded conditions

**DOI:** 10.3389/fpls.2026.1873609

**Published:** 2026-09-08

**Authors:** Happiness P. Mbelle, Shilpa Manhas, Akida Ignas Meya, Frank Stephano Mabagala, Chinmaya Sahoo, Ankit Saini, Regan Makarius Nyoni, Mohamed Yehia Abouleish, Ahmed Elbeltagi

**Affiliations:** 1 Department of Agronomy, School of Agriculture, Lovely Professional University Phagwara, Phagwara, Punjab, India; 2 Department of Sustainable Development, The Nelson Mandela African Institution of Science and Technology (NM-AIST), Arusha, Tanzania; 3 Department of Soil Science, Ukiriguru Research Institute, Mwanza, Tanzania; 4 Department of Agronomy, Dr. Rajendra Prasad Central Agricultural University, Pusa, Bihar, India; 5 Department of Agronomy Dr Khem Singh Gill Akal College of Agriculture, Eternal University, Baru Sahib, Sirmaur, India; 6 Tanzania Coffee Board, Ministry of Agriculture, Moshi, Kilimanjaro, Tanzania; 7 College of Arts and Sciences, Department of Biology, Chemistry and Environmental Sciences, American University of Sharjah, Sharjah, United Arab Emirates; 8 Sustainable Agriculture Research Group, College of Arts and Sciences, American University of Sharjah, Sharjah, United Arab Emirates; 9 College of Agriculture, University of Al Dhaid, Al Dhaid, Sharjah, United Arab Emirates

**Keywords:** biochar, dry direct-seeded rice, integrated nutrient management, rice varieties, soil fertility, sustainable agriculture

## Abstract

**Introduction:**

The increasing need for food, which results from fast population growth, requires agricultural methods that can produce more crops while protecting soil health. Biochar has become an effective soil enhancer that boosts soil fertility and nutrient retention while increasing crop productivity.

**Methods:**

The study examined the effects of nutrient management with biochar on the growth performance of direct-seeded rice in the Bagamoyo irrigation project during the cropping period of 2025. The split-plot design was used with three replicates and a split-plot ANOVA and linear mixed effect models to assess the integration of biochar and NPK from maize straw in three rice varieties (TXD 306, TANRICE 1, and KHAODUCKMALL) under multi-season conditions, with a p<0.05 mean separation and a Tukey HSD.

**Results:**

The study results demonstrate that biochar application constituted the only element that produced a statistically significant impact on crop height at harvest level measurement (p = 0.0135), while all other factors, including variety and their interactions, showed no significant impact. The two most important factors for sample discrimination were Fe and Zn, while TN and K followed behind. The PCA results showed that PC1 explained most of the variation through its strong links to Fe and Zn and TN, while PC2 showed primary connections to P and K and Mn. The biplot showed three distinct clusters, which scientists identified with 95 confidence with confidence ellipses. The 50% biochar plus 50% NPK treatment outperformed the control and sole fertilizer treatments because it produced taller plants, although the varieties showed minor differences in their plant response.

**Discussion:**

The study results show that biochar combined with mineral fertilizer produced greater tiller manure yields when compared to the control treatment. The results demonstrate that combining biochar with inorganic fertilizers enhances rice growth and productivity in drought-prone direct-seeding conditions while also improving soil fertility management.

## 1 Introduction

The global population has been growing steadily in recent decades, putting significant pressure on food production systems. The world population is projected to reach 9.7 billion by 2050, which will create a higher need for food, feed, and all other agricultural products, according to Iasmin et al., 2025. Global agricultural production must increase by nearly 60% because of increasing population food needs, which creates a serious problem for both sustainable development and food security (Berners-Lee et al., 2018; Valavanidis, 2023). The growing population demands improved crop productivity together with better resource efficiency and sustainable land management methods to secure future food supplies (Giller et al., 2022; Alexandratos and Bruinsma, 2012).

Rice (*Oryza sativa L.*) serves as a fundamental crop, which sustains worldwide food security efforts. More than half the global population depends on rice as their main source of food, particularly in Asian countries and developing nations where rice provides their primary energy needs (Bandumula, 2018; Darko et al., 2025; Elbeltagi et al., 2026). Future food demands will require a significant increase in global agricultural production (Berners-Lee et al., 2018; Valavanidis, 2023).

The rising need for rice because of the increasing population requires major changes to rice production methods in order to meet the upcoming demand (Asma et al., 2023). The productivity of agricultural systems faces challenges because farmers use intensive farming methods that reduce soil health, deplete nutrients, and harm the environment. Farmers use chemical fertilizers to enhance soil fertility and boost crop production to meet rising rice demand (Shah and Wu, 2019).

Plants require essential nutrients, which fertilizers supply through nitrogen (N), phosphorus (P), and potassium (K) according to Sinha and Tandon, 2020. Farmers in most agricultural systems have adopted chemical fertilizers as their primary method of crop cultivation. The use of these fertilizers boosts crop productivity at first, but their excessive application causes multiple issues that include soil degradation, nutrient imbalance, and a decrease in soil microbial activity and environmental contamination, according to Sabina et al., 2025. The high cost of chemical fertilizers, which mainly come from basic mineral nutrients, makes them unaffordable for small farmers in many developing countries, according to Bisht and Chauhan, 2020 and Saha et al., 2024.

The excessive application of chemical fertilizers results in nutrient leaching, soil acidification, and soil organic matter loss, which causes long-term damage to soil health and agricultural productivity, according to Pahalvi et al., 2021. The agricultural sector requires sustainable soil amendments that enhance soil fertility and decrease the need for artificial fertilizers. The implementation of biochar presents an effective method to enhance soil quality and boost crop yield. Biochar is created through the pyrolysis process, which transforms organic biomass materials like crop residues into a carbon-rich material that requires oxygen-limited conditions for production (Xu et al., 2026; Kong et al., 2026). The material has been of considerable interest in recent years because it can improve soil properties by influencing physical, chemical, and biological properties of the soil. Biochar creates a material with a highly porous structure and a large surface area, which allows for better oxygen flow in soil, as well as better water retention and nutrient retention. These characteristics support the root development process, which also increases crop growth and productivity according to Alkharabsheh et al., 2021 and Huang et al., 2013. Multiple research investigations demonstrate that applying biochar leads to better soil fertility and improved crop cultivation because biochar enhances soil organic carbon content, nutrient accessibility, and microbial activity. The addition of biochar to agricultural soils results in better nutrient preservation, which leads to increased crop yields. The recent meta-analysis demonstrates that rice farmers who apply biochar achieve approximately 10 percent higher yields than those who do not treat their fields because of better soil structure, nutrient access, and nitrogen management (Jaswal et al., 2025). Biochar increases soil organic carbon content and total nitrogen, which leads to better soil fertility that supports plant development (Kapoor et al., 2022; Zhang et al., 2024). The research shows that biochar application enhances soil characteristics while establishing optimal conditions for rice cultivation. The research shows that applying biochar results in higher microbial activity and better soil nutrient management while decreasing salinity and other soil stressors, which leads to improved plant growth and increased rice output (Agegnehu et al., 2017; Kapoor et al., 2022).

Researchers have established that biochar supports environmental sustainability because its permanent carbon composition allows soil carbon to remain intact for extended durations. This process decreases climate change effects because it lowers atmospheric carbon dioxide while enhancing soil quality and agricultural efficiency (Lian and Xing, 2017; Luo et al., 2023). Biochar functions as an environmentally safe soil amendment, which supports sustainable agricultural practices. The sustainability of modern agriculture faces increasing threats because intensive farming methods lead to soil degradation.

The combination of continuous tillage and excessive chemical fertilizer applications has caused a decline in soil organic matter and imbalanced nutrient levels, nutrient leaching, and soil degradation, according to Liu et al., 2015 and Lian and Xing, 2017. The degradation process leads to diminished soil capacity that prevents healthy crop growth and decreases agricultural productivity throughout an extended period. The rice-growing systems experience their most serious challenges because farmers keep cultivating their fields while removing nutrients without proper soil fertility restoration methods, according to Singh et al., 2025.

The agricultural landscapes create substantial amounts of crop waste, which farmers usually burn in their fields to create air pollution and greenhouse gas emissions while losing precious organic materials (Smil, 1999; Bhuvaneshwari et al., 2019). The combustion process can be avoided as these residues are transformed into stable and valuable organic compounds, including biochar. Biochar is a carbon-rich material that results from a pyrolysis process that converts crop residues into carbon-rich material by reducing the exposure to oxygen. Its reputation as a soil improver has gained worldwide recognition, as biochar improves soil health and helps plants retain their nutrients, while promoting sustainable farming practices. Biochar improves the physical, chemical, and biological characteristics of soil, leading to better soil structure and water retention and better nutrient availability, and serves as a permanent solution for carbon sequestration, thus helping to mitigate climate change (Lorenz and Lal, 2014; Elkhlifi et al., 2023). Biochar can be combined with chemical fertilizers to improve nutrient use efficiency, enabling crops such as rice to absorb nutrients more effectively and reducing nutrient losses from fertilizers (Ning et al., 2022; An et al., 2022). The integrated approach provides a sustainable method to enhance both agricultural output and soil nutritional content. The benefits of biochar use have led to a growing appreciation of its effects on soil health and on the growth and productivity of rice in emerging systems of water-intensive and labor-intensive cultivation, which are more efficient than traditional paddy systems. The economic effects of biochar usage together with chemical fertilizers need to be studied because this knowledge will help farmers accept biochar as a viable agricultural solution (Sandhu et al., 2021). Researchers have studied biochar applications in many studies, but they mainly focused on general crop responses that failed to recognize the different nutrient uptake and growth patterns that exist between rice varieties. The limited number of long-term field studies on biochar’s residual effects makes it impossible to understand its long-term advantages for soil health (Rehman et al., 2025).

The optimal application rates and types of biochar rice cultivars remain largely undetermined, which creates uncertainty in the practical agricultural advice that farmers receive. These knowledge gaps highlight the need for targeted research to assess the variability in rice’s biochar response under field conditions (Danso Ofori et al., 2025). The development of sustainable land management strategies requires researchers to address existing deficiencies because these strategies will boost rice production while enhancing soil health and supporting Tanzania’s food security and soil conservation goals. The study has current relevance because it addresses pressing issues. The study aims to assess the impact of biochar on soil health in accordance with established requirements. The study aims to assess the various combinations of biochar and fertilizer treatments in order to determine their economic feasibility. Research is examining the effects of biochar application on the growth and yield performance of rice plants grown by direct sowing. The study will examine how the use of biochar affects nutrient uptake and rice quality in different varieties. The research study shows sustainable practices in the use of biochar as a soil improver, resulting in higher rice yields and improved soil health and environmental sustainability.

This study aims to investigate the effects of biochar on soil health, evaluate the synergistic effects of biochar–fertilizer combinations, and quantify their influence on the growth and yield performance of rice grown under direct-seeding conditions.

## 2 Materials and methods

### 2.1 Study area and experimental site

**Figure 1:**
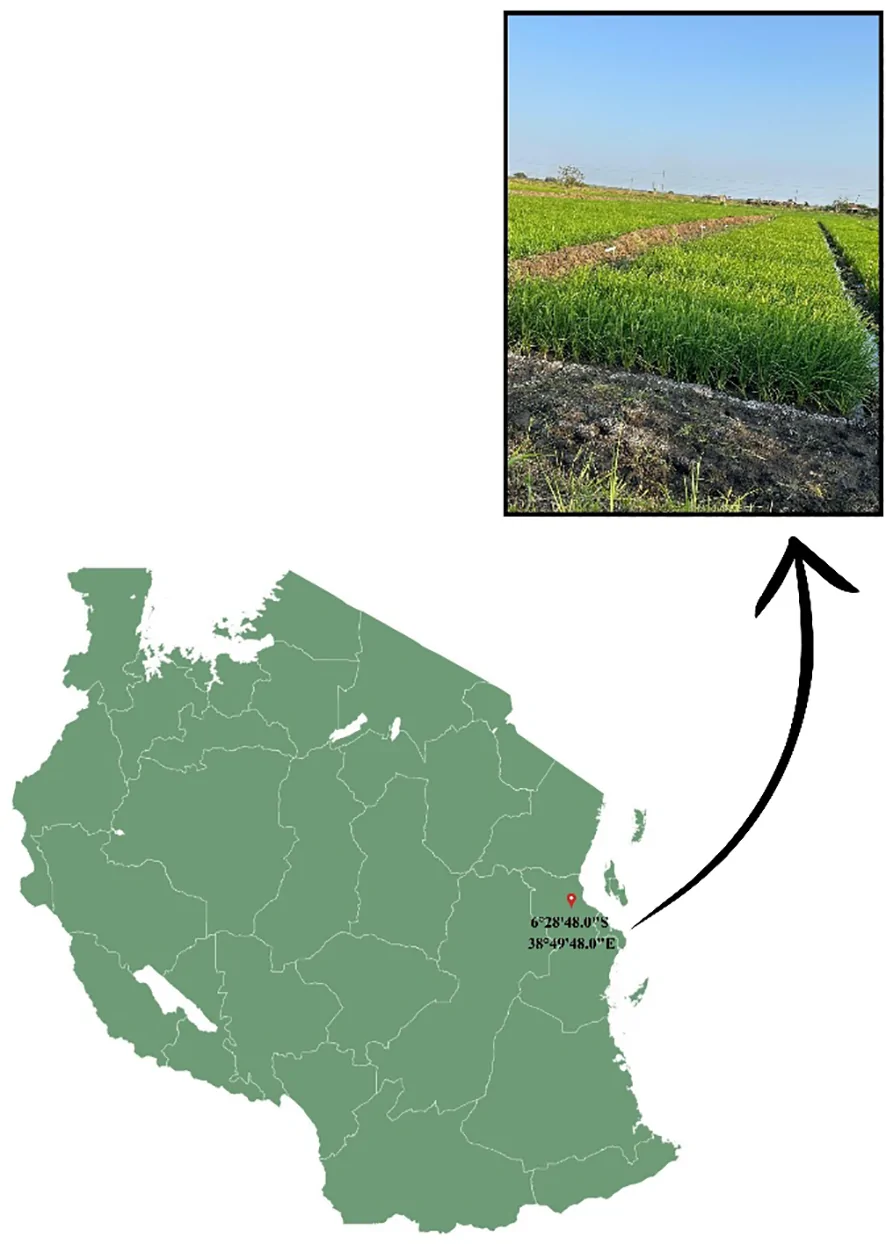
Representative view of expiremental site at the Bagamoyo Irrigation Project, Tanzania, showing irrigated rice plots under field trial conditions.

The field trial was conducted at the Bagamoyo irrigation project from 22 June to 5 November 2025 during the 2025 cropping season (**Figure 1**). The study area is in Tanzania’s coastal zone, which serves as an important region for rice cultivation under both irrigated and semi-irrigated agricultural methods. The region experiences tropical weather patterns, which bring two rainy seasons and maintain moderate temperatures and high humidity throughout the year. The experiment tested dry direct-seeded rice cultivation, which farmers now use because it helps them save water and labor while keeping their rice production rates steady.

The field trial was conducted at the Bagamoyo irrigation project in coastal Tanzania during the 2025 cropping season, which lasted from 22 June to 5 November 2025. The region experiences a tropical climate, which features two distinct rainy seasons that include extended rainfall from March to May and brief rainfall from October to December. The area experiences average temperatures between 25 and 32 degrees Celsius, together with high humidity levels.

The site represents a common lowland rice production system that exists within both irrigated and semi-irrigated agricultural areas. The research investigated DDSR, which is an innovative farming method that decreases water usage and labor requirements while producing competitive agricultural results and reducing financial expenses. The researchers conducted composite soil sampling, which covered the 0 to 20 cm depth range, to obtain basic physicochemical data through analysis of soil samples that included pH values, organic carbon content, total nitrogen, phosphorus availability, and soil texture.

### 2.2 Experimental design and treatments

The experiment used a split-plot design, which had three replicates as its testing method. The proposal has been selected to assess the interaction between rice varieties and nutrient management strategies that include biochar and inorganic fertilizers.

The experiment was set up as a partitioned design with three replications. Each plot was 4 m x 3 m (12 m 2) with a spacing between the plants of 20 cm x 20 cm. Boundary lines have been kept around each plot to minimize edge effects and to prevent nutrient interference between treatments. Standard agronomic practices were consistently followed in all practices. Manual weeding was applied twice at 20 and 40 DAS to control the competition from weeds. The pest and disease management has been carried out using the recommended Integrated Pest Management (IPM) strategies. Irrigation was applied as necessary to maintain optimum soil moisture under drought-conditioned direct-seeded conditions and to ensure that water stress is avoided during critical growth stages. These consistent management practices have ensured that the differences observed are mainly due to the effects of the treatment.

### 2.3 Main plot factor: Rice varieties

Three rice varieties commonly cultivated in Tanzania were used as the main plot treatments: TXD 306 (SARO), TANRICE1, and KHAODUCKMALL. The researchers chose these varieties because they wanted to study how different growth patterns, nutrient absorption capabilities, and harvesting outcomes would affect their results.

### 2.4 Sub-plot factor: biochar and fertilizer treatments

Five nutrient management treatments were assigned to the sub-plots:

T_1_: Absolute control (no biochar and no fertilizer)
T_2_: 100% biochar
T_3_: 100% recommended NPK fertilizer
T_4_: 50% biochar + 50% NPK fertilizer
T_5_: 75% biochar + 25% NPK fertilizer

Each treatment combination was replicated three times to improve statistical reliability and minimize experimental error.

The recommended rice fertilizer dose (RDF) was applied on the basis of the blanket fertilizer recommendation, which is equivalent to a N:P_2_O_5_:K_2_O ratio of 120:60:40 kg of ha^-^¹. The 100 percent NPK treatment was fully reimbursed by the RDF, while the 50 percent and 25 percent NPK treatments were reimbursed by 60:30:20 kg ha^-^¹ and 30:15:10 kg ha^-^¹, respectively. Nitrogen was applied in three equally divided doses: at sowing, during active tillage (25–30 DAS), and at the panicle-opening phase in order to minimize losses and improve uptake efficiency. At the time of sowing, phosphorus and potassium were applied as basal doses. Biochar has been incorporated into the soil before sowing in order to allow sufficient interaction with soil nutrients to occur.

### 2.5 Biochar production and preparation

The experiment used biochar that was produced from maize straw, which local farms provided as their agricultural waste. The biochar production process employed slow pyrolysis technology through a drum kiln method, which exists as an economical method for creating agricultural biochar. The experiment used maize biochar, which originated from maize straw residues that underwent slow pyrolysis in a drum kiln. The maize straw collection process began with farmers gathering the straw, which they then air-dried to decrease its water content. The team conducted a combustion experiment by filling metal drums with dry residues, which they burned under controlled oxygen conditions that were restricted to create conditions for the pyrolysis process. The material required cooling after carbonization until it reached a proper temperature, which allowed staff to remove it from the furnace. The process generated biochar, which operators crushed and ground to produce uniform particle sizes that farmers could use as soil amendment products. The researchers stored maize biochar in airtight containers until its field application to prevent moisture contamination, which would compromise its quality. The biochar used in this study was characterized by a pH of 8.2, a cation exchange capacity (CEC) of 32 cmol kg^-^¹, a specific surface area of 85 m² g^-^¹, total carbon (C) content of 65%, total nitrogen (N) content of 0.8%, total phosphorus (P) content of 0.4%, and total potassium (K) content of 1.2%.

### 2.4 Biochar characterization and application rates

Biochar used in this study was obtained from maize straw feedstock and produced by slow pyrolysis by the traditional drum-based method. The pyrolysis was maintained at a maximum carbonization temperature of approximately 450 °C for three hours to ensure complete thermochemical transformation. Biochar was used in the relevant sub-plots at a total of 10 t ha^-1^ (for a 100 percent biochar treatment), with adjusted proportional inputs of 5 t ha^-1^ (50 percent biochar) and 7 t ha^-1^ (75% biochar).

### 2.5 Data analysis

The data were analyzed by ANOVA using a split-plot design with three replicates. The main plots were allocated to rice varieties (V_1_: TXD 306, V_2_: TANRICE 1, V_3_: KHAODUCKMALL) and the sub-plots to combinations of biochar and NPK fertilizer. Data from two seasons were pooled, and Bartlett testing confirmed the homogeneity of the variance. A mixed-effect model was used with season as a random variable and treatment of variety and nutrients as fixed effects, including their interactions. For the sequence growth data, a repeated-measures ANOVA was applied with the growth phase as the internal control factor. The analyses were carried out with R and with IBM SPSS (v26). Significant differences were reported with p<0.05, and the means were stratified by the Tukey HSD test. A PCA was used to visualize the treatment effects in a multivariate manner.

## 3 Results

The interaction between rice varieties and biochar-fertilizer treatment has been shown to have a clear and significant impact on the height of the plants, the yield of the crop and the yield of the grain (**Table 1**). Overall, the integration of biochar with inorganic fertilizer consistently improved crop performance compared to a single application or control, although the magnitude of the response varied from variety to variety. The plant height was highest in TXD 306 and TANRICE 1 below 100 percent NPK and in combination with biochar, with a value in some cases above 100 cm, while KHAODUCKMALL had a relatively lower value, especially when treated with biochar alone and with controls (**Table 1**). Similarly, tillage production peaked at TXD 306 below 100 percent NPK (29.30 tillers hill-1) and integrated treatment maintained a high tillage intensity for all varieties, indicating a beneficial synergy between biochar and mineral fertilizers (**Table 1**). Grain yields responded most strongly to the integration of nutrients. The highest yield was observed in TANRICE 1 under the 75-75–25 biochar + NPK combination (6.92 t ha^-^¹), closely followed by TXD 306 under the 75-75-50–75 combination (6.41 t ha^-^¹ and 6.39 t ha^-^¹, respectively) (**Table 1**). By contrast, the control treatment consistently produced the lowest yield of all varieties, i.e. from 2.18 to 2.81 t ha^-^ (**Table 1**). Although biochar alone improved yields compared to control, it was still less efficient than integrated nutrient management (**Table 1**).

**Table 1:** Interaction effects of rice varieties and biochar–fertilizer treatments on plant height, tillers, and grain yield.

Variety	Treatment	Plant height (cm)	Tillers (no. hill^-^¹)	Grain yield (t ha^-^¹)
KHAODUCKMALL	T1	86.37 ± 1.9^b^	23.57 ± 1.0^a^	5.97 ± 0.23^a^
	T2	91.63 ± 2.0^ab^	23.33 ± 1.1^a^	5.46 ± 0.27^a^
	T3	96.27 ± 2.1^a^	24.33 ± 1.2^a^	5.36 ± 0.25^a^
	T4	79.63 ± 1.8^c^	17.67 ± 0.9^c^	4.90 ± 0.21^b^
	T5	77.27 ± 1.7^c^	21.77 ± 0.8^b^	2.47 ± 0.18^c^
TANRICE 1	T1	94.33 ± 2.0^ab^	23.33 ± 1.1^ab^	6.92 ± 0.24^a^
	T2	96.80 ± 2.1^a^	24.67 ± 1.2^a^	5.10 ± 0.28^a^
	T3	100.33 ± 2.4^a^	23.67 ± 1.1^ab^	5.68 ± 0.26^a^
	T4	96.97 ± 2.2^a^	22.87 ± 1.0^b^	5.05 ± 0.22^b^
	T5	84.63 ± 1.8^b^	21.86 ± 0.9^c^	2.81 ± 0.19^c^
TXD 306	T1	89.50 ± 2.0^b^	25.77 ± 1.1^b^	6.41 ± 0.25^a^
	T2	101.43 ± 2.4^a^	27.68 ± 1.2^a^	6.39 ± 0.30^a^
	T3	101.33 ± 2.5^a^	29.30 ± 1.3^a^	5.61 ± 0.27^b^
	T4	85.17 ± 1.9^b^	24.37 ± 1.1^b^	4.80 ± 0.20^c^
	T5	76.57 ± 1.6^c^	20.00 ± 0.8^c^	2.18 ± 0.17^d^

Values followed by the same letter within a column are not significantly different at p < 0.05 according to the Least Significant Difference (LSD) test.

Values are mean ± SE. Means within each variety followed by the same letter are not significantly different at p < 0.05 according to Fisher’s LSD test.

The research results demonstrate that integrated nutrient management through biochar and inorganic fertilizer blending leads to better crop results while providing a sustainable solution that decreases dependence on chemical fertilizers.

### 3.1 Effects of biochar and variety on grain yield and yield components

The analysis showed that biochar application produced a strong impact on grain yields, which reached statistical significance at p-value 0.0005 and brought about yield improvements to all tested treatment groups according to **Table 1** results. The main effect of the variances showed no statistical significance because the p-value reached 0.0976, yet researchers observed a slight trend. The varieties responded to biochar treatment in an identical fashion because the interaction between variety and biochar showed no statistical significance at p-value 0.8076. The two factors of variety and biochar treatment brought about major changes to the number of fields which farmers ploughed during each harvest period. The study found that genetic variation between varieties directly affected tiller production while biochar treatment achieved an additional increase in this production. The interaction effect showed no statistical significance because the p-value reached 0.3630 (**Table 2**). Biochar application showed a direct impact on crop height at harvest, while neither variety nor their combined effects produced any significant outcome. Biochar treatment produced a very strong effect on panicle numbers at harvest, which scientists measured with a p-value of 0.0034, whereas neither the variety effect nor the interaction produced any results. The LAI showed significant changes because both variety and biochar treatments implemented genetic modifications and soil alterations, which influenced canopy growth (**Table 2**).

**Table 2:** Summary of split-plot ANOVA showing the effects of variety (main plot), biochar application (sub-plot), and their interaction on yield and selected growth and yield components.

Variable	Variety (main plot)	Biochar (sub plot)	Interaction (Var × Bchr)
Yield (t/ha)	0.0976 ns	0.0005 ***	0.8076 ns
Tiller No. (Harvest)	0.0428 *	0.0203 *	0.3630 ns
Plant Height (Harvest)	0.4055 ns	0.0135 *	0.3927 ns
Panicle No. (Harvest)	0.1151 ns	0.0034 **	0.7402 ns
LAI	0.0409 *	0.0203 *	0.3887 ns
Dry Matter (30 DAS)	0.0443 *	0.3873 ns	0.4230 ns
Days to 50% Flowering	0.1191 ns	0.2421 ns	0.6072 ns
Harvest Index (HI)	0.0587 ns	0.3369 ns	0.6481 ns
Grains per Panicle	0.5821 ns	0.7564 ns	0.5368 ns
Panicle Length	0.8159 ns	0.4043 ns	0.2953 ns

Significance levels are indicated as ns (not significant), *p < 0.05, **p < 0.01, and ***p < 0.001.

The research showed that different plant varieties affected dry matter accumulation at 30 days after sowing because of their different growing characteristics. The research demonstrates that early productivity of the plants resulted from their genetic traits instead of any soil improvement techniques. The days to 50% flowering and the harvest index were not significantly influenced by variety, biochar, or their interaction, suggesting a limited effect of the treatments on crop phenology and biomass distribution. The research found that all tested factors failed to produce any significant impact on both grains per panicle and panicle length, which revealed that these traits display stable patterns throughout different time periods.

The ANOVA results (**Table 2**) showed that biochar use had a very significant effect (p<0.001) on rice yield, indicating that biochar use had a significant effect on rice productivity. Rice variety had no impact on yield because it showed no statistical significance at (p > 0.05), which proved that nutrient management was the main factor affecting yield performance in the study. The variety and biochar interaction did not have any effect on yield or most parameters, which showed that all varieties responded to biochar in the same way. The two tested plant varieties and biochar treatment produced significant effects on tiller number and leaf area index measurement, which demonstrated how genetic properties and soil alterations work together to determine plant growth. Plant height and panicle numbers showed significant changes through biochar application, which demonstrated that biochar improves plant growth and development of reproductive parts. The variety produced strong effects on early growth, which scientists measured through dry matter accumulation during the first 30 days after sowing, because the genetic makeup of the different varieties determined how vigorously their seedlings would grow. Biochar now stands as the primary factor that controls various agricultural traits.

### 3.2 Principal component analysis of the variability of nutrient grains

**Figure 2:**
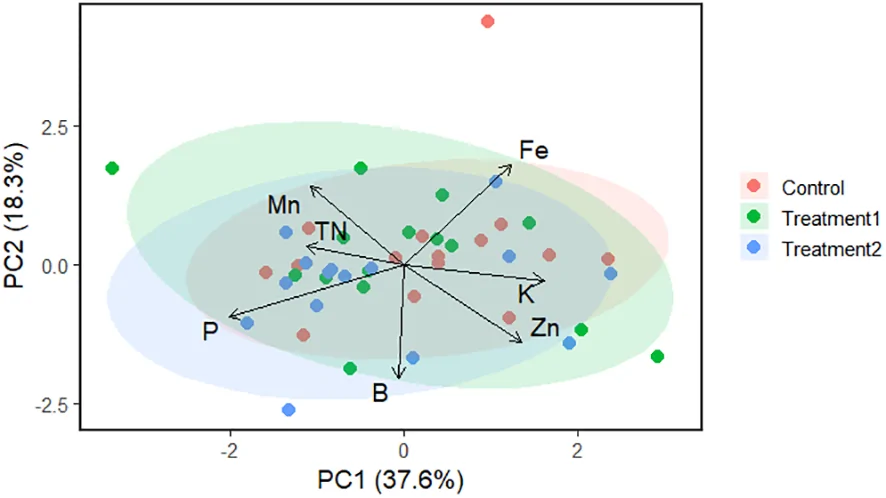
PCA biplot of nutrient variables for the treatment group in the experiment. The main components, Dim1 and Dim2, account for 37.6% and 18.3% of the total variance, respectively. The vectors indicate the direction and strength of the loading of TN, P, K, Mn, Fe, Zn, and B, respectively.

The multidimensional dataset was reduced by principal component analysis to its main components, which accounted for most of the total variance. The first PC1 accounted for the largest portion of variation, which showed a strong relationship with Fe, Zn, and TN. The second PC2 showed a main relationship with P K and Mn. The PCA biplot (**Figure 2**) displayed three sample clusters, which were separated by 95% confidence ellipses that showed distinct multivariate groupings. The vector lengths indicated that Fe and Zn were the main factors that distinguished between the samples, while TN and K followed as secondary contributors. The pattern of clustering shows that rice reference nutrient differences occur because of combined macro and micronutrient effects instead of one nutrient effect.

### 3.3 Plant height dynamics under integrated biochar and mineral fertilizer application

**Figure 3:**
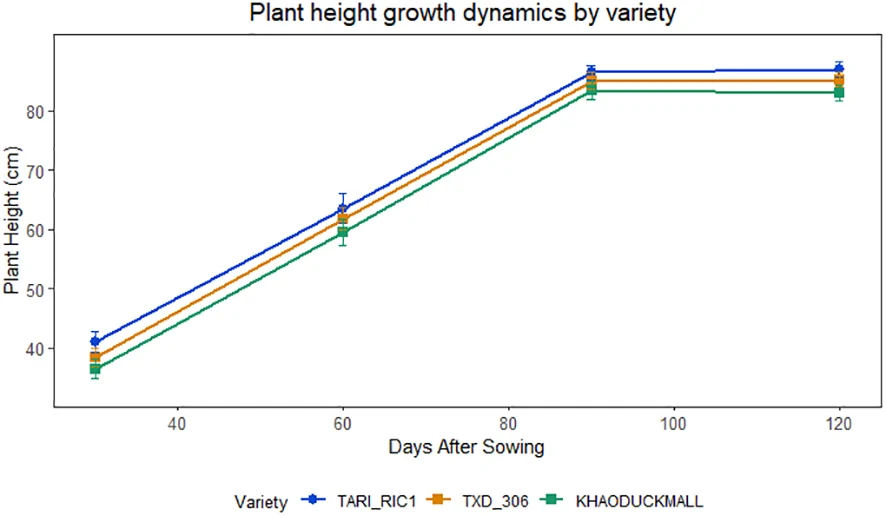
Varietal response and plant height growth dynamics of TARI_RIC1, TXD_306, and KHAODUCKMALL at 30, 60, 90, and 120 days after sowing (DAS).Each data point is the mean ± S.E. of three replications.

**Figure 4:**
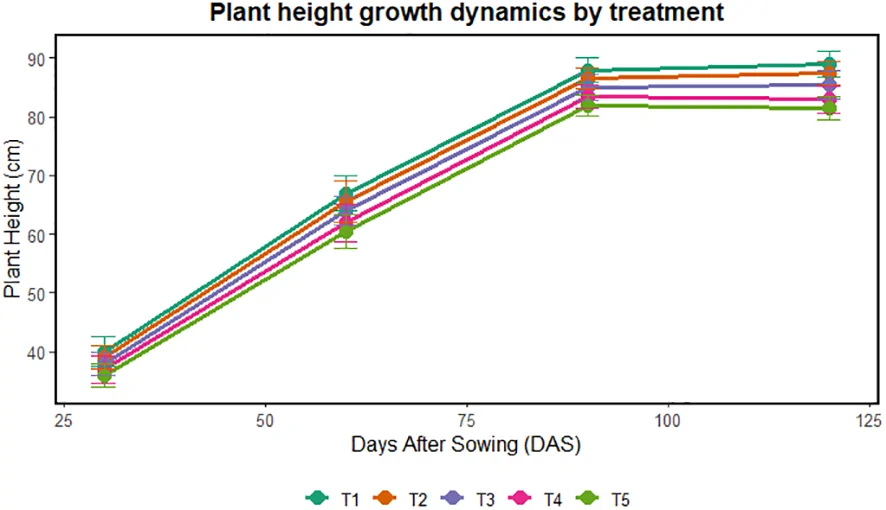
Effect of biochar and NPK fertilizer combinations on rice plant height at 30, 60, 90, and 120 days after sowing (DAS), illustrating the comparative performance of integrated nutrient treatments. Each data point is the mean ± S.E. of three replications.

The plant height for all studied varieties showed continuous growth throughout all biochar treatments, which followed a sigmoid growth curve that showed rapid plant development from 30 to 90 days after sowing and reached its maximum growth at 120 days after sowing (**Figure 3**). The rice varieties TARI_RIC1, TXD_306, and KHAODUCKMALI showed equal crop establishment throughout the first growth period from 30 to 60 days after sowing because no significant differences (p > 0.05) existed between these two groups. The treatment effects increased in strength during the period that started at 90 days after sowing and continued until harvest at 120 days after sowing. The 50% Biochar + 50% NPK treatment produced taller plants than both the control (Absolute) and sole fertilizer treatments, even though different plant varieties responded to treatments with different outcomes. Plant height differences between treatments became visible at 90 days after sowing, and at 120 days after sowing, the combined biochar–NPK treatment produced better or equal plant height results than other treatment options. The results demonstrate that integrated nutrient management exercises stronger control over plant development during reproductive and grain-filling stages than during the initial vegetative growth period (**Figure 4**).

### 3.4 Tiller number dynamics under biochar treatments

**Figure 5:**
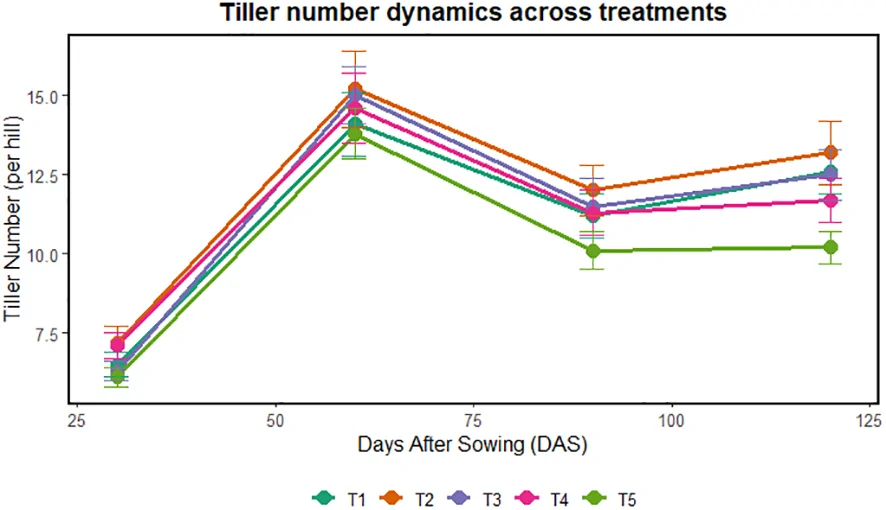
Effect of biochar and NPK fertilizer treatments on tiller number dynamics of rice from 30 to 90 days after sowing (DAS). Each data point is the mean ± S.E. of three replications.

The number of tilled rice plants follows a typical physiological pattern, with a rapid increase in the early growth stages and a decrease in the maturing period (**Figure 5**). The number of tractors increased sharply from 30 days after sowing to 60 DAS, reached a maximum at 60 DAS, then decreased to 90 DAS due to natural tractor mortality. Rice plants decrease their weaker tillers because they need to channel their resources toward the most productive tillers, which will develop into panicles. The 75 percent Biochar + 25 percent NPK treatment and the 100 percent NPK treatment produced the most tilled beds during the peak stage, which resulted in about 16–17 tilled beds per plant at 60 DAS. The absolute control approach produced the least number of tillers during the entire growth period, reaching its highest count of approximately 11 tillers.

**Figure 6:**
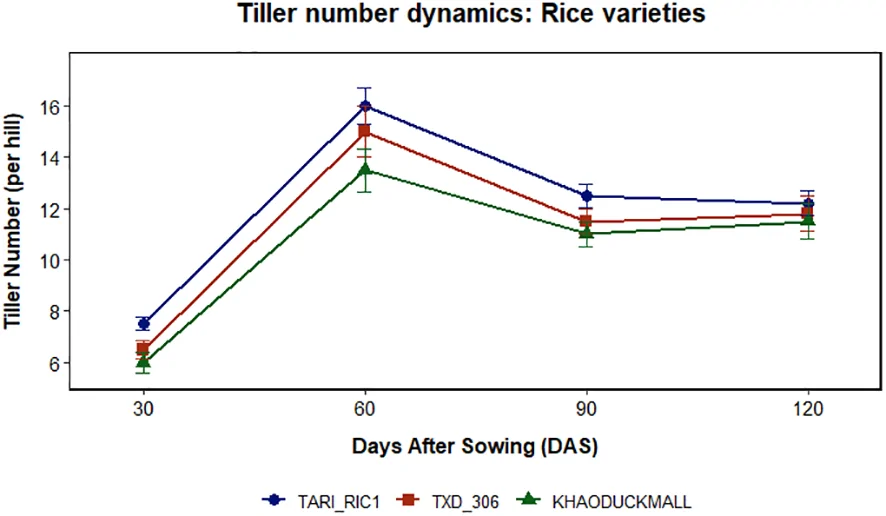
Tiller dynamics of three rice varieties (TXD 306, TARI RIC1, and KHAODUCKMALL) during the growing period of 30, 60, 90, and 120 days. Each data point is the mean ± S.E. of three replications.

The results demonstrate that biochar application, together with mineral fertilizer, produced higher tiller manure output than the control group (**Figure 6**). The increased tilling yields show that biochar improves soil conditions and nutrient availability, which results in better plant growth and increased tilling. The different rice varieties displayed distinct differences in their ability to handle tillage operations. The TXD 306 plant at 60 DAS showed the maximum tiller count, which demonstrated its capacity for vegetative development. TARI RIC1 displayed moderate tillage performance throughout the growing season, while its operational capacity remained steady until the harvest period. KHAODUCKMALL produced the lowest number of tillage workers during its growth phase. The TXD 306 system demonstrates superior tillage capacity, which results in higher yield potential because productive tillage represents a fundamental element that affects rice grain output.

### 3.5 Dry matter dynamics under biochar treatments

**Figure 7:**
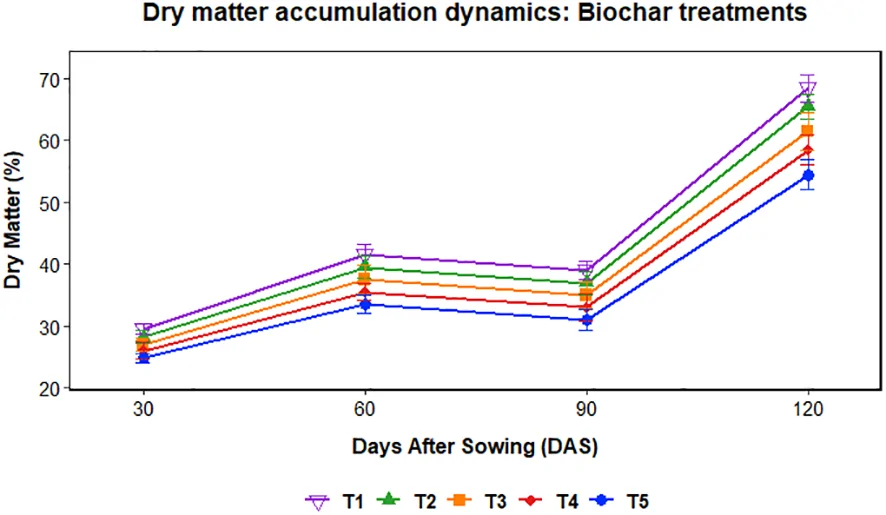
Effect of biochar and NPK fertilizer treatments on dry matter accumulation of rice from 30 to 120 days after sowing (DAS). Each data point is the mean ± S.E. of three replications.

The rice plants developed dry matter according to a standard growth pattern, which continued until the end of their growing season, from 30 days after sowing until 120 days after sowing. The early vegetative phase, between 30 and 60 days after sowing, showed no treatment differences because all treatment groups developed dry matter at the same rate. The research results showed that nutrient management practices only affected biomass production during the first growing period (**Figure 7**). The research results showed different treatment effects, which became evident during the reproductive and maturation phase that occurred between 90 and 120 days after planting. At physiological maturity (120 DAS), absolute control showed the lowest dry matter accumulation, indicating a reduction in biomass production in the absence of fertiliser inputs. The later growth stages of dry matter development showed better results with fertilisation than other methods. The 100 NPK treatment and the 50 NPK with Biochar treatment produced the highest dry matter results at 120 DAS, which showed their superior ability to produce biomass and their better grain filling capability than all other treatments. The control treatments showed clear differences from the fertiliser treatments because the nutrient changes led to substantial increases in both crop growth and biomass retention. The results demonstrate that biochar, together with mineral fertilizers, helps rice plants maintain biomass growth while postponing their initial aging process.

**Figure 8:**
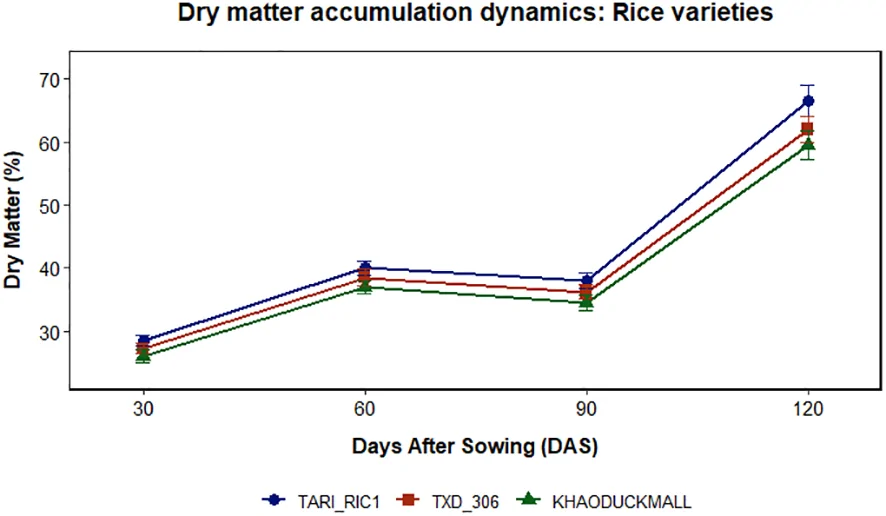
Dry matter accumulation patterns of three rice varieties measured at 30, 60, 90, and 120 days after sowing (DAS). Each data point is the mean ± S.E. of three replications.

Rice varieties displayed different patterns of dry matter accumulation (**Figure 8**). The growth pattern of all varieties showed a sigmoidal shape because they first developed their biomass in their early stages, and then they experienced fast growth during their reproductive phase before their growth reached its final stage. TXD 306 variety maintained a steady pattern of dry matter accumulation throughout the entire growing period and reached its peak biomass weight at 120 DAS. TARI RIC1 showed a sharp increase in the accumulation of dry matter from 90 to 120 DAS, which indicates a strong biomass increase during the filling phase of the grain. KHAODUCKMALL showed stable growth throughout its development process, but it produced less dry matter than other varieties when they reached their maturity stage. The higher dry-matter accumulation observed in TXD 306 produces higher yields because the relationship between biomass production and rice yield potential shows a strong connection.

## 4 Discussion

### 4.1 Effects of biochar and variety on grain yield and yield components

Biochar enhances crop production through its ability to enhance soil physicochemical characteristics and maintain soil nutrients and water resources, according to Yadav et al. (2018) and Alkharabsheh et al. (2021). The study demonstrated that biochar application produced significant positive results for rice yield because the experimental results showed that biochar functions as an effective soil enhancer (p = 0.0005; **Table 1**). The study found that varietal changes did not produce measurable effects on crop yields (p = 0.0976), and the study results showed no interactive effects between varietals and biochar (p = 0.8076), which indicated that different genetic backgrounds showed similar yield responses to biochar application. Joseph et al. (2020) discovered that biochar application consistently raised crop yields for multiple plant species, which matched the findings of Jeffery et al. (2017) and Yimer and Tarnawa (2025), who showed that increased nutrient availability with soil moisture preservation led to better crop yields. The results demonstrate that biochar produces consistent positive effects on crop yields, which occur regardless of the crop variety being tested.

The enhancement of cereal yield results from better yield components, which include increases in tiller count and panicle count, according to research by Kamiji et al. (2011), Mmbando (2025) and Asefa (2019). The study results showed that biochar treatment created a statistically significant impact through its effects on both till number at harvest time and panicle count (p=0.0203 and p=0.0034, respectively, **Table 1**), whereas the varietal effect showed statistical significance for till number (p=0.0428). The research results indicate that biochar acted as a direct source that helped plants create productive tillers and panicles that ultimately resulted in higher grain production. The research results match those found by Vijayaraghavan (2019), who reported that biochar treatment led to higher biomass and yield components, and by Liu et al. (2021), who discovered that better soil fertility led to greater plant growth. The research findings align with previous studies, which demonstrate that biomass-mediated yield increase results mainly from improved plant growth and sink development (Wang et al., 2026).

The genetic differences among different crop types lead to variations in their initial growth, their ability to develop a canopy, and their capacity to grow biomass (Richards et al., 2002, Sidahmed et al., 2025; Zhang et al., 2026). The research demonstrated that the variety had a major impact on dry matter accumulation at 30 DAS (p = 0.0443; **Table 1**), but biochar showed no impact during this initial period (p = 0.3873). The leaf area index (LAI) measurements showed a significant impact from both the plant variety (p = 0.0409) and the biochar application (p = 0.0203). The results indicate that genetic traits control initial growth rates while biochar application results in better canopy development during the subsequent growth period. Rice and other cereal varieties showed similar differences in early biomass production, which resulted from seedling vigor that depended on their genotypic makeup (Balaout 2026; Khan et al., 2024; Mohapatra et al., 2025). The study found no significant differences between varieties regarding final grain yield, which indicates that biochar treatment helped compensate for the early growth differences observed during the experiment.

The genetic factors lead to early development of traits, while soil modification impacts show stronger effects on final productivity outcomes. The harvest index and overall yield stability are determined by the phenotypic and reproductive characteristics of plants according to research conducted by Cao et al., (2025), Sarwar et al., (2025) and Pratap et al., (2025). The study found that variety, biochar, and their interaction did not have any impact on the number of grains per panicle and panicle length, and days to 50 percent flowering and harvest index, according to **Table 1**. The research shows that biochar does not impact reproductive efficiency or phenological development, but it enhances yield through vegetative growth. Khan et al., 2022 found that biochar increases biomass and yields without affecting flowering time or harvest index, which confirms their previous research results. The study found that increased resource capacity through biomass and tillage brought more yield increases than changes in reproductive distribution. The research results show that biochar applications increase crop production through improved plant development and crop yield traits, which maintain their effects across different plant varieties. The genetic variations between individuals showed impacts on their growth patterns, yet biochar application proved to be the primary factor that determined their ultimate yield results. The study results support earlier studies while showing that biochar functions as an effective, sustainable soil management approach; however, extended studies across multiple environmental conditions need to take place to validate its applicability in different situations.

### 4.2 Principal component analysis of the variability of nutrient grains

The research showed that TN, P, and K had a positive connection, which demonstrated that plant nutrient absorption and environmental conditions worked together during their growth process. Nitrogen functions as a growth stimulant for plant roots while it supports membrane transport processes and boosts enzyme production that controls phosphorus-based biochemical reactions. The studies of Cao et al. and Wang et al. show that an adequate nitrogen supply results in improved phosphorus absorption because it helps roots develop better and increases the body’s phosphorus absorption needs. The element potassium exists as a critical component of nitrogen metabolism through its function in activating enzymes and creating proteins. The PCA analysis established three macronutrients, which showed physiological connections because TN, P, and K appeared together in the same principal component. Cereal crops show similar positive connections among their main nutrients because better nitrogen fertilization leads to more root development, which results in higher phosphorus and potassium absorption through increased biomass growth.

Researchers found a positive correlation between Fe and Zn measurements, which indicated that both minerals move through the body in a synchronized manner. Previous studies by Welch and Graham (2004) reported that iron and zinc share common transporters, such as ZIP transporters, and that they employ similar chelation mechanisms during grain loading. The biofortification methods used in their research typically result in simultaneous increases of both micronutrients. The PCA analysis shows that Fe and Zn vectors maintain proximity, which demonstrates their synchronized distribution throughout the ecosystem. Mn developed a weaker relationship with other micronutrients from the study because it followed a different pathway for regulation. Manganese uptake patterns show more variation than iron and zinc because soil redox conditions and plant genotype have strong effects on manganese absorption. The PCA results demonstrate that plants accumulate macronutrients through metabolic coordination while they use both shared and specific regulatory pathways to develop micronutrients during grain development.

### 4.3 Plant height dynamics under integrated biochar and mineral fertilizer application

The observed sigmoid growth trend shows that rice plants follow the normal growth pattern, which shows their vegetative development reaching its fastest point during their middle growth phase and then maintaining its speed until they attain maturity. The early growth phase study results show no major differences, which indicates that plant development during this stage depended more on seed quality and soil fertility than on treatment effects. The 50 percent biochar plus 50 percent NPK treatment at later times showed more height growth because biochar application enhanced nutrient absorption and soil conditions, and natural organisms became more active. Biochar enhances cation exchange capacity together with its ability to retain nutrients and water, which results in improved nitrogen accessibility and uptake efficiency according to Pühringer (2016). The study conducted by KC and Mahat (2024) found that plants achieved higher growth and biomass production when they received both biochar and mineral fertilizer compared to those that received only mineral fertilizer. Integrated nutrient management methods maintain baseline nutrient release while minimizing nutrient runoff, which leads to better crop yield outcomes during their reproductive phase. The results of our research confirm existing scientific studies, which demonstrate that using biochar as a partial replacement for inorganic fertilizers leads to improved plant development while decreasing chemical fertilizer requirements.

The higher performance of the 50–50 biochar + 50–50 NPK treatment is attributed to synergistic interactions between organic and inorganic sources of nutrients under direct seeding. Biochar improves the retention of moisture in soil, porosity, and exchange capacity of cations, thus increasing nutrient retention and reducing leaching. Combined with NPK, it ensures both immediate and permanent availability of nutrients throughout the growing season. This balanced supply is supported by an improved leaf area index, crop yields, and biomass accumulation, which in turn leads to higher grain yields. Compared to single applications, the integrated approach optimizes the efficiency of nutrient use and soil fertility, and is therefore the most efficient and sustainable nutrient management strategy.

### 4.4 Tiller number dynamics under biochar treatments

The research demonstrated that rice tiller dynamics followed the standard growth pattern, which showed rapid tiller development during the vegetative period and produced a decline in tiller growth after the peak tiller count because of natural tiller death (Mohapatra et al, 2025). The plant experiences resource allocation to its productive panicles because of this reduction, which happens when non-productive tillers are removed. Rice production systems show identical tillage patterns, which reach their highest level between 45 and 65 days after sowing, according to specific crop variety and agricultural management method. The 75 percent biochar and 25 percent NPK combination resulted in increased tiller production because biochar enhanced soil fertility, together with better nutrient accessibility. Biochar enhances soil properties by providing better nutrient retention ability, cation exchange capacity, and aeration and water retention capacity, which together support plant development and soil fertility creation. The research conducted by Faloye et al. (2017) and Arif et al. (2017) found that biochar combined with inorganic fertilizers led to increased tiller bed production and biomass growth.

The research demonstrates that 75 percent biochar and 25 percent NPK exhibit the same agricultural productivity as 100 percent NPK because they produce identical crop yields. The genetic potential of TXD 306 to develop vegetative growth showed strong evidence through its highest tiller worm production, which matched the findings of Shahzad et al. (2019). TARI RIC1 displayed consistent tiller maintenance capabilities, whereas KHAODUCKMALL demonstrated reduced tiller maintenance, which results in its lower profitability. The research findings demonstrate that combining biochar with reduced NPK fertilizer application results in higher tiller production while sustaining rice agricultural output.

### 4.5 Dry matter dynamics under biochar treatments

The accumulation of dry matter serves as a crucial measurement for determining crop development because it shows how well plants transform sunlight energy into their biomass (Lambers et al., 2019). In this study, dry matter demonstrated a typical sigmoid growth pattern, which started with gradual growth, reached rapid expansion during the reproductive phase, and ended with stabilization at maturity. The early vegetative phase (30–60 DAS) showed no treatment differences because the plants depended mainly on their seed reserves and the nutrients present in the soil. The absolute control condition produced the least dry matter growth at maturity, which indicates that proper nutrient management is essential for achieving consistent biomass and grain production (Chen et al., 2026; Kumara et al., 2026).

The treatment using 50 percent biochar and 50 percent NPK produced dry matter results that matched the results of 100 percent NPK, thus showing that biochar can serve as a partial replacement for mineral fertilizers. Biochar enhances soil properties through its ability to maintain nutrients and water and to boost microbial activity, which results in improved crop development and increased biomass production. Arif et al. (2017); Tammeorg et al. (2014), and Gu et al. (2025) reported similar research results, which showed that biochar delivered better crop growth when combined with inorganic fertilizers. The highest biomass production occurred under TXD 306, which showed different growth patterns among its various plant types. The TARI RIC1 exhibited fast biomass development during grain filling, but KHAODUCKMALL showed lower biomass, which resulted in its reduced crop output. The research findings demonstrate that biochar mixed with lower NPK levels can sustain biomass growth while improving rice crop yield.

### 4.6 Effect of biochar and fertilizer on rice yield

Biochar application significantly increased rice yield (p < 0.001), consistent with previous studies attributing yield improvements to enhanced soil structure, increased cation exchange capacity (CEC), and greater microbial activity (Zhang et al., 2025; Yan et al., 2026). Rice variety had no significant effect on grain yield, and the absence of a significant variety × biochar interaction suggests that biochar-based nutrient management is broadly applicable across different rice genotypes (Singh et al., 2026; López Espinoza, 2026). Unlike most previous studies conducted under irrigated conditions, this research evaluated biochar–NPK integration under tropical dry direct-seeded rice, demonstrating the effectiveness of defined biochar–NPK ratios (50:50 and 75:25) for optimizing nutrient management across multiple rice varieties.

### 4.7 Limitations, implications, and future outlook

This study was limited to one growing season and did not allow for an assessment of the long-term effects of biochar on the soil characteristics and the productivity of the rice. Moreover, due to differences in soil and climate conditions, the findings may not be directly transferable to other agro-ecological areas. Future research should focus on long-term field-testing, economic feasibility, microbial interactions between biochar and soil and carbon sequestration in order to better determine the sustainability and scalability of nutrient management with biochar.

## 5 Conclusion

This study showed that the integration of biochar with inorganic fertilizer significantly improved the growth and yield of rice from direct sowing under drought conditions. Among the treatments assessed, the combination of 50% biochar + 50% NPK consistently produced the highest yields, leaf area and harvest indices, showing its potential as an efficient integrated nutrient management strategy. The results show that the partial substitution of inorganic fertilizers by biochar can increase nutrient efficiency while reducing reliance on chemical fertilizers and thus promote the production of sustainable and climate-smart rice. Although the study was carried out over one growing season, the results provide a strong basis for the recommendation of the inclusion of biochar-NPK in dry direct-seeded rice systems. Further multi-seasonal and multi-location studies are needed to confirm its long-term agronomic, economic and environmental benefits, especially in small-scale agricultural systems.

## Data Availability

The original contributions presented in the study are included in the article/supplementary material. Further inquiries can be directed to the corresponding authors.
